# Analysis of patients with head injuries: A retrospective study

**DOI:** 10.6026/973206300212210

**Published:** 2025-07-31

**Authors:** Vineet Mandrah, Sandeep Thakre, Kishor Uikey, Dileep Dandotiya

**Affiliations:** 1 Department of Surgery, CIMS, Chhindwara, Madhya Pradesh, India; 2 Department of Orthopaedics, Government Medical College, Seoni, Madhya Pradesh, India; 3 Department of Community Medicine, CIMS, Chhindwara, Madhya Pradesh, India

**Keywords:** Glasgow coma scale (GCS), head injury, neurosurgical, traumatic brain injury

## Abstract

Head injury with subsequent Traumatic brain injury (TBI) is a major source of morbidity and mortality and presents a challenge. A retrospective study was conducted on patients hospitalized to the Department of Surgery having traumatic brain damage that ranges from moderate to severe (GCS <12/15). In all, 1291 individuals participated, 411 had moderate head injuries (9/15 GCS score) and 240 had a severe brain injury (GCS score of less than 8/15). It was noted that neurosurgical intervention was necessary for less than 32% of patients in an emergency.

## Background:

Traumatic head injuries are a major public health-related issue throughout the world and the commonest cause of death in the 15-44 year age group [1]. At the global level, it is estimated that the annual incidence of Traumatic Brain Injuries (TBIs) is 200 per 100,000 per year and mortality is 20 per 100,000 per year [2]. Most of the deaths and disability are due to Head injury and the rate of mortality and disability increases with the severity of the trauma [3]. In India, brain trauma damage poses a substantial risk to the health of the public that causes young, productive members of our society to die, be injured, or become disabled. In an epidemiological study undertaken in Bangalore, it was revealed that the incidence was 150/1,00,000, the mortality rate was 20/1,00,000 and the case fatality rate was 10%, respectively [4]. The number of traffic accidents and assaults is considerably higher in the young adult population compared with the other age groups. Mortality in Head injury patients mostly arises from preventable conditions, and the young adult population seems to be the most affected group [5]. In developing countries like India, road traffic accidents are increasing day by day due to industrialization and urbanization, which leads to head injuries [6]. Head injury cases usually come to the emergency department of hospitals that have diagnostic imaging facilities. Glasgow coma scale is used for determining the severity of head injury and the type of intervention required [7]. The first stage of cerebral injury after TBI is characterized by direct tissue damage with impaired regulation of cerebral blood flow and metabolism. The second stage of the patho-physiological cascade is characterized by terminal membrane depolarization along with excessive release of excitatory neurotransmitters (*i.e.*, glutamate, aspartate), activation of N-methyl-D-aspartate, α-amino-3-hydroxy-5-methyl-4-isoxazolpropionate and voltage-dependent Ca2+- and Na+-channels [2]. Between January 2011 and December 2013, the Department of Surgery at the multispecialty Maharaja Yashwant Rao (MYH) Hospital in Indore saw a total of 24,477 cases. Out of these, 2,822 were classified as head injury cases, which call for a deeper epidemiological analysis given their considerable effects on public health and how resources are allocated [8]. Therefore, it is of interest to retrospectively analyze head injury cases admitted to MY Hospital, Indore, between 2011 and 2013, to evaluate epidemiological trends over three years, assess the availability and adequacy of treatment facilities along with causes of any delays and determine the burden of disease in terms of morbidity and mortality.

## Materials and Methods:

Maharaja Yashwant Rao (MY) Hospital, which is linked with MGM Medical College in Indore, carried out a retrospective analysis using hospital records of patients who were admitted with head injuries to the Department of Surgery from January 2011 to December 2013. This study looked at all consecutive cases, regardless of their severity, to evaluate epidemiological trends and outcomes. The study patients having a GCS of greater than 13/15 are excluded. Similarly, individuals who were not hospitalized or relocated to the surgical department were not included in this study. Data characteristics include the patient's age, sex, reason for the accident, any further injuries outside brain traumas, alcohol use, any surgeries or medical treatments received earlier.

## Results and Discussion:

When the cases were classified according to etiology, incidents involving road traffic collisions constituted the majority: 49.2% in 2011, 49.2 % in 2012 and 43% in 2013. Falls from heights are another etiological factor in head injury cases, constituting 22.7% in 2011, 25.5% in 2012 and 25.3% in 2013. Incidents of aggression made up 24.5% in 2011, 23.3% in 2012 and 26.4% in 2013. Athletic injury accounted for 8% in 2011, 10% in 2012 and 12% in 2013. Other injuries, such as train accidents, animal injuries and falls from trees and poles, constituted 2.6% in 2011, 3.9% in 2012 and 4.33% in 2013 (Table 1 - see PDF). In 2011, the mild head injury group GCS 13-15 constituted 49.7% of cases, the moderate head trauma GCS 9-12 group made up 31.3% of cases and the Critical cranial trauma group constituted 19% of cases. In 2012, the mild head injury group accounted for 49.3% of cases; the moderate head trauma group represented 32.5% of cases and the Critical cranial trauma group made up 18.2% of cases. In 2013, the mild head injury group constituted 49.7% of cases, the group with moderate head trauma accounted for 31.8% of cases and the Critical cranial trauma group constituted 18.6% of cases (Table 2 - see PDF). Road Traffic Accidents (Motor vehicle crashes) were the leading cause of head injuries in total, followed by assault and falls. RTAs accounted for 28.5% of head injuries during the third decade of one's life, while assaults accounted for 37.3% (Table 3 - see PDF). About 56% of the treated patients were referred from other hospitals and 44% were non-referred cases. Out of 844 cases of severe head injury, 63.6% were referred from other hospitals, whereas 36.8% of cases were directly admitted. Out of 413 cases of moderate head injury, 65.3 5 were referred from other hospitals, whereas 34.6% were directly admitted. In the mild head injury group comprising 1535 patients, 50.2% were directly admitted and 49.8% were referred from other hospitals (Table 4 - see PDF). In one of the studies, this was reported that the majority of patients improved to a GCS of 15 within one month; those who were admitted showed significant neurological recovery [9]. In 2011, burr hole operations accounted for 21%, craniotomy accounted for 32.3% and depressed fracture operations accounted for 46.8%. In 2012, burr whole operations accounted for 19% cases; craniotomy for mass effect lesions constituted 34.3% and depressed fractures constituted 46.7% cases. In 2013, burr holes accounted for 21.6% cases, craniotomy for mass lesions 34.4% and depressed fractures constituted 44% cases (Table 5 - see PDF). Road traffic accidents made up 1291 of the 2822 incidents in the study; of these, 51% involved individuals on two-wheeled vehicles, 31% involved individuals inside four-wheeled vehicles and 18% involved individuals traveling on foot. Of the 610 patients with mild head injuries, Sixty-three percent of the individuals surveyed were riders of motorcycles or scooters, 19% were four-wheeler occupants, Eighteen percent of the individuals involved were pedestrians. 39% of the 281 instances in the moderate head injury group had individuals riding on two-wheeled vehicles, 35% involved occupants of four-wheeled vehicles and 26% involved pedestrians. Of the 400 instances in the severe head injury group, 40% involved two-wheelers, 46% involved four-wheelers and 14% included pedestrians (Table 6 - see PDF). Out of the total number of admissions, 29.9% belonged to the severe head injury group. 14.6% belonged to the moderate head injury group and 55.4% belonged to the mild head injury group. Out of the total number of deaths, 69% occurred in the severe head injury group and 31% belonged to the moderate head injury group. There were no deaths in the mild head injury group (Table 7 - see PDF). In high-income nations, the primary factor contributing to mortality and impairment at the moment is cerebral trauma. By 2020, it is expected to overtake all other causes of disability and mortality worldwide [10]. Every year, around 6 million people in India suffer from TBI [11]. In India, the entire cost of traffic-related injuries is thought to be around 3% of GDP. The case fatality rate was 10%, the death rate was 20/1,000,000 and the incidence was 150/1,000,000, according to the epidemiological study conducted in Bangalore [12, 13-14]. The following study, conducted by this institute between 2011 and 2013, reflects the recent workload in neurosurgery. Teasdale *et al.* devised the GCS Scoring system to classify the severity of head injuries [15]. In the study of 2822 cases, 29.9% belonged to the severe head injury group according to GCS score, about 14.6 % belonged to moderate head injury group and 55.5 % belonged to mild head injury group (Table 8 - see PDF). Clinical guidelines give specific parameters for management, ventilation, prophylaxis, temperature control, nutritional support, *etc.*, to manage traumatic brain injury, although further research on cytotoxic and inflammatory pathways is required for management of secondary injury following head trauma [16]. In another retrospective study conducted on patients admitted to multidisciplinary ICU between 2021 and 2023, it was found that 73.6% of patients had TBI due to RTA. Mortality was high among patients with higher Rotterdam computed tomography (CT) scores and mechanical ventilation following TBI was required in 84.2% of patients. Mortality following TBI was high, with 46.05% of all patients dying [17].

## Conclusion:

In patients with trauma, Head injuries are most common and assessing their severity is very important for applying appropriate treatment strategies. It is noted that head injury ratio in males and females was found to be 5:1. Further, head trauma occurred in automobile accidents among all age groups.

## Funding:

Nil

## Author's contribution:

VM: Conceptualized, designed the study, data collection, literature search, intellectual content definition, collected data and manuscript writing. ST: Screened the included articles and helped in manuscript writing. KU: Preparing and reviewing the manuscript and supervising all phases of the research process. DD worked with VM to prepare the manuscript and conduct data analyses. The final manuscript was read and approved by all writers.
